# Risk of acute kidney injury following contrast-enhanced CT or MRI in a cohort of 3061 hospitalized children in China

**DOI:** 10.1186/s12887-024-04875-z

**Published:** 2024-06-19

**Authors:** Chen Wang, Chao Zhang, Jihang Sun, Ruohua Yan, Xiaohang Liu, Lulu Jia, Xiaoxia Peng

**Affiliations:** 1 Center for Clinical Epidemiology and Evidence-based Medicine, Beijing Children’s Hospital, Capital Medical University, National Center for Children Health, No.56 Nanlishi Road, Beijing, 100045 China; 2 Department of Radiology, Beijing Children’s Hospital, Capital Medical University, National Center for Children’s Health, Beijing, China; 3 Department of Pharmacy, Beijing Children’s Hospital, Capital Medical University, National Center for Children’s Health, No.56 Nanlishi Road, Beijing, 100045 China

**Keywords:** Contrast media, Acute kidney injury, Computed tomography, Magnetic resonance imaging, Risk ratio

## Abstract

**Objectives:**

To compare the risk of acute kidney injury (AKI) between hospitalized children who received intravenous contrast media for imaging examinations and those who did not.

**Methods:**

This retrospective cohort study enrolled patients aged 0–18 years with serum creatinine levels before and after imaging examinations from 2015 to 2020 at Beijing Children’s Hospital. Participants were classified into an exposure group or a control group. Log-binomial regression analysis was used to estimate the adjusted risk ratio (aRR) value for the association between exposure to contrast media and consequential AKI. After which, inverse probability treatment weighting was used to reduce systematic differences in baseline characteristics among the groups. Moreover, subgroup and sensitivity analyses were performed. Finally, multivariate logistic regression analysis was performed to identify risk factors for pediatric AKI.

**Results:**

In total, 3061 pediatric patients were included in the analyses (median age, 4.5 [IQR, 1.3–8.9] years, 1760 males). According the KDIGO definition of AKI, the incidence of AKI in the exposure group, and the control group were 7.4% and 6.5%, respectively; furthermore, the aRR was 1.35 (95% CI: 1.31–1.39). In patients underwent CT, the risk of AKI in the exposure group of contrast media increased compared with the control group and the aRR was 1.39 (95% CI: 1.09–1.78). However, it is not observed in patients underwent MRI (aRR: 1.36; 95% CI: 0.96–1.95). According to our subgroup analysis of pediatric patients aged ≥ 2 years (aRR: 1.38; 95% CI: 1.05–1.82) and sensitivity analysis (aRR: 1.32, 95% CI: 1.08–1.61), the risk of AKI in the exposure group was greater than that in the control group. An increased risk to exposure to contrast media was seen in females (aRR: 1.41, 95% CI: 1.05–1.89) rather than males (aRR: 1.30, 95% CI: 0.99–1.70). According to the multivariate logistic regression analyses, the baseline eGFR (OR: 1.02; 95% CI: 1.01–1.03) and comorbidities (OR: 2.97; 95% CI: 1.89–4.65) were risk factors, while age (OR: 0.87; 95% CI: 0.84–0.91) was a protective factor against AKI.

**Conclusion:**

The evidence from the present study suggested that the increased risk of AKI in hospitalized children induced by intravascular contrast should not be ignored.

**Supplementary Information:**

The online version contains supplementary material available at 10.1186/s12887-024-04875-z.

## Introduction

Contrast media are essential for enhanced imaging examinations, such as iodine-based contrast mediums used in computed tomography (CT) and gadolinium-based contrast agents used in magnetic resonance imaging (MRI), which helps physicians to obtain indispensable information [1]. However, the impacts of intravenous iodine-based contrast on renal function, including renal tubular epithelial cells damage and hemodynamic perturbations induced by contrast media, are still a research hotspot [2–4]. Although initially believed to be without major adverse effects, gadolinium-based contrast agents was demonstrated to cause nephrogenic systemic fibrosis in patients with abnormal renal function [5]; moreover, a recent study indicated that exposure to Gadolinium was related to both necrosis and apoptosis of proximal tubular epithelium which led to kidney injury after Gadolinium exposure [6, 7]. A retrospective study found that sequential exposure to iodine- and gadolinium-based contrast media on the same admission was a risk factors for post-contrast acute kidney injury (AKI) [1].

Contrast-associated acute kidney injury (CA-AKI) is characterized by a decrease in kidney function that occurs within several days after the intravascular administration of contrast material during imaging examination [1, 2], which is similar with post-contrast acute kidney injury [1, 8] and includes contrast-induced AKI (CI-AKI) [9]. The diagnosis of CI-AKI requires a causal temporality between contrast media and AKI as well as excluding other possible causes [10]. Therefore, the concept of CI-AKI has rarely been used in recent years due to the complexity of causal inference [9]. Due to the different definitions mentioned above, the incidence of AKI after exposure to contrast media in pediatric patients has been reported to range from 1.4 to 35.0% [11–17].

In recent years, the implementation of preventive care has resulted in lower rates of CA-AKI, such as hydration administration which was a well-known preventive measure for CA-AKI in adults [18]. A meta-analysis based on adult participants suggested that contrast media does not increase the risk for CA-AKI [19]. One study recently reported that the risk of AKI due to contrast media was overestimated [20]. However, it is difficult to simply administer conduct intravenous hydration administration in the pediatric patients due to their young age, light weight, and poor compliance of invasive procedures. Nonetheless, the impacts of contrast media on renal function in pediatric patients remain a hot topic of research, especially for children aged < 2 years old with immature renal function [3, 4]. Recently, Calle-Toro et al. reported an increased risk of AKI in pediatric patients with an estimated glomerular filtration rate (eGFR) ≥ 60 mL/min/1.73 m^2^ when they were exposed to contrast media, which was inconsistent with the findings of previous studies regarding pediatric CA-AKI [13, 15]. Therefore, it is necessary to conduct further investigations on the risk of AKI in pediatric patients related to contrast media exposure.

Based on the routine electronic medical record data of Beijing Children’s Hospital in China, this retrospective cohort study aimed to compare the risk of AKI in hospitalized children who received intravenous contrast media for CT or MRI examinations with that in those without exposure to contrast media, these findings could provide further evidence for the risks of CA-AKI to help clinicians and radiologists to weigh the ratio of benefits and harms of contrast media in the pediatric setting.

## Methods

### Participants

**Fig. 1:**
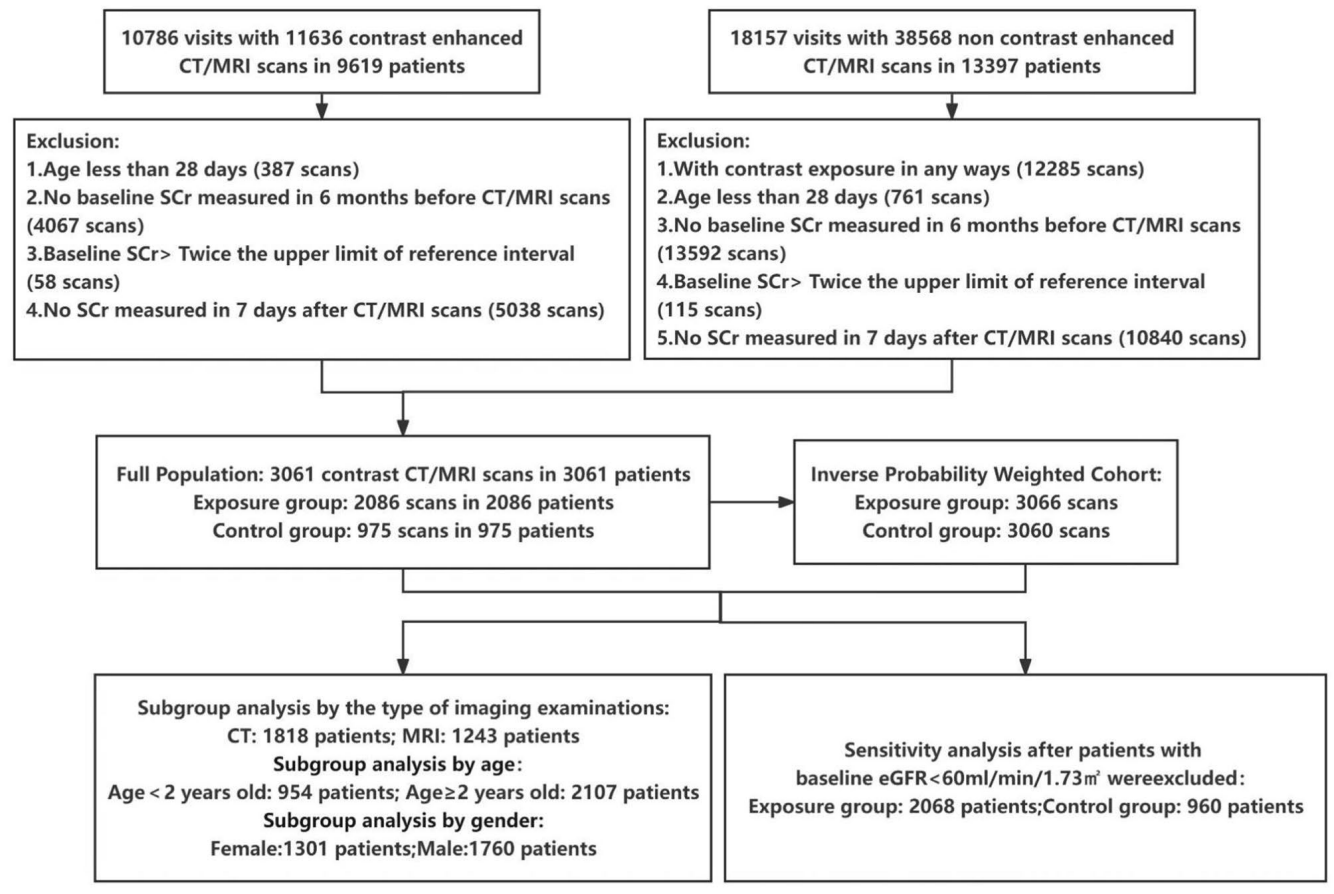
Flowchart showing the process used to select patients. eGFR = estimated Glomerular Filtration Rate, SCr = Serum Creatinine

This retrospective cohort included pediatric patients aged 0–18 years with serum creatinine (SCr) measurements before and after CT/MRI at Beijing Children’s Hospital. The routine electronic medical record data were extracted from January 2015 to December 2020 in the hospital information system and laboratory information management system. The protocol was approved by the Institutional Review Board of Beijing Children’s Hospital, Capital Medical University (approval number: [2022]-E-207-Y), with a waiver of informed consent. The study included all hospitalized patients (age range, 0–18 years) whose SCr was available before and after undergoing a CT/MRI scan. The patients were classified into groups according to exposure to contrast media. The exposure group was defined as patients with contrast-enhanced CT/MRI, while the control group was defined as the patients with non-contrast-enhanced CT/MRI. The exclusion criteria were as follows: (1) had a history of AKI, dialysis or renal replacement therapy during hospitalization; (2) were aged less than 28 days due to no consensus was reached about the reference intervals of SCr in China; (3) lacked important information, such as age, CT/MRI scans time, and baseline SCr (the most recent value at 6 months before CT/MRI scans); (4) had a baseline SCr level more than twice the upper limit of the reference intervals according to age and sex groups [21] and had already met the stage 2 AKI according to the Kidney Disease Improving Global Outcomes (KDIGO) criteria for AKI [22]; (5) had their SCr level not measured within 7 days after CT/MRI. Furthermore, only the first record of contrast enhanced imaging examination was included if the patients had multiple contrast-enhanced imaging examination during hospitalization. The flow chart of participants in this retrospective cohort is shown in Fig. 1.

### Definition of contrast media exposure

The exposure was defined as the intravascular exposure of contrast material during enhanced CT/MRI examination in hospitalized children. In Beijing Children’s hospital, the pediatrician made the decision whether to use the contrast agent. All contrast-enhanced CT examinations performed on patients were performed with low-osmolality iodinated contrast media or iso-osmotic iodine contrast media. The dose of the above contrast media was calculated based on the body weight iodine contrast media concentration and examination (listed in Supplemental Table 1). The contrast media used in MRI examinations was gadolinium-based contrast agent which was administered intravenously based on the patient’s weight. The detailed information regarding contrast material administration in CT/MRI scans is provided in Supplemental Method 1.

### Definition of CA-AKI

The primary outcome was the incidence of AKI. AKI was defined on the basis of changes in the SCr level in all study patients according to the KDIGO definition (i.e., compared with the baseline SCr, an increase in the SCr level ≥ 0.3 mg/dL [26.5 µmol/L] or ≥ 50% within 7 days after CT/MRI examinations) [22]. The stage of AKI was also defined by the KDIGO criteria [22]. The baseline SCr was defined as the most recent value obtained 6 months before CT/MRI, which represent the routine level of renal function in pediatric patients before imaging examinations. The SCr concentration for diagnosing AKI was defined as the highest value tested within 7 days after CT/MRI. The AKI was diagnosed according to the rapid decrease of kidney function within 7 days after imaging examinations compared with the routine level. The urine output criteria were not used in this research due to the unavailability of data on urine output.

### Definition of other covariables

All covariables were collected before the CT/MRI scans including the following: age, sex, department of hospitalization (intensive care units [ICU] admission or not), comorbidities (such as diabetes mellitus [including type 1 and type 2 diabetes mellitus], hypertension, kidney disease, sepsis, cardiac failure, and respiratory failure), laboratory examination values of SCr, baseline eGFR, and the number of nephrotoxicity drugs taken during hospitalization (the detailed list of nephrotoxicity drugs in our research is shown in Supplemental Table 2). The eGFR was calculated by the FAS-equation, which was developed by the Pottel term in Belgium and is based on the SCr and the age of pediatric patients [23].

### Statistical analysis

First, nonnormally distributed continuous variables are described by using medians and interquartile ranges (IQRs), whereas categorical variables are described by counts and percentages. The Mann-Whitney U test and chi-square test or Fisher’s exact test (when conditions for the chi-square test were not met) were performed to assess differences in continuous and categorical variables between the exposure group and the control group, respectively (Supplemental Table 3 ‒ 6).

Second, the propensity score (PS) was calculated by multivariate logistic regression to adjust for potential confounders including age, sex, baseline eGFR, ICU admission, comorbidities and the number of nephrotoxicity drugs [24]. The distribution of propensity score in exposure group and control group is shown in Supplemental 3. 1. Then, we calculated the inverse probability of the exposure weight of each participant with or without exposure to contrast media in terms of the PS. The weights were defined as 1/PS for participants exposed to contrast media and 1/(1-PS) for participants not exposed to contrast media [25]. The inverse probability of treatment weighting (IPTW) methods was used to improve the balance between exposure group and control group and reduce systematic differences in baseline characteristics among participants with or without exposure to contrast media induced by covariates [25]. A standardized mean difference (SMD) less than 0.1 indicated that covariate imbalance between groups could be ignored (Supplemental Fig. 2) [26]. In addition, all weights generated by the PS model were used in our analysis for not detecting extremely large weight values in the IPTW distributions (Supplemental Fig. 3).

Third, the log-binomial regression analysis was used to estimate the association between exposure to contrast media and AKI and calculate the crude (cRR) of unweighted cohort and adjusted risk ratios (aRR) of IPTW cohort. Moreover, subgroup analyses were performed in terms of the type of imaging examination (CT or MRI), age (defined by age < 2 years or ≥ 2 years old), and sex (defined by female or male). A sensitivity analysis was performed after the pediatric patients with eGFR < 60 mL/min/1.73 m^2^ were excluded.

**Fig. 2:**
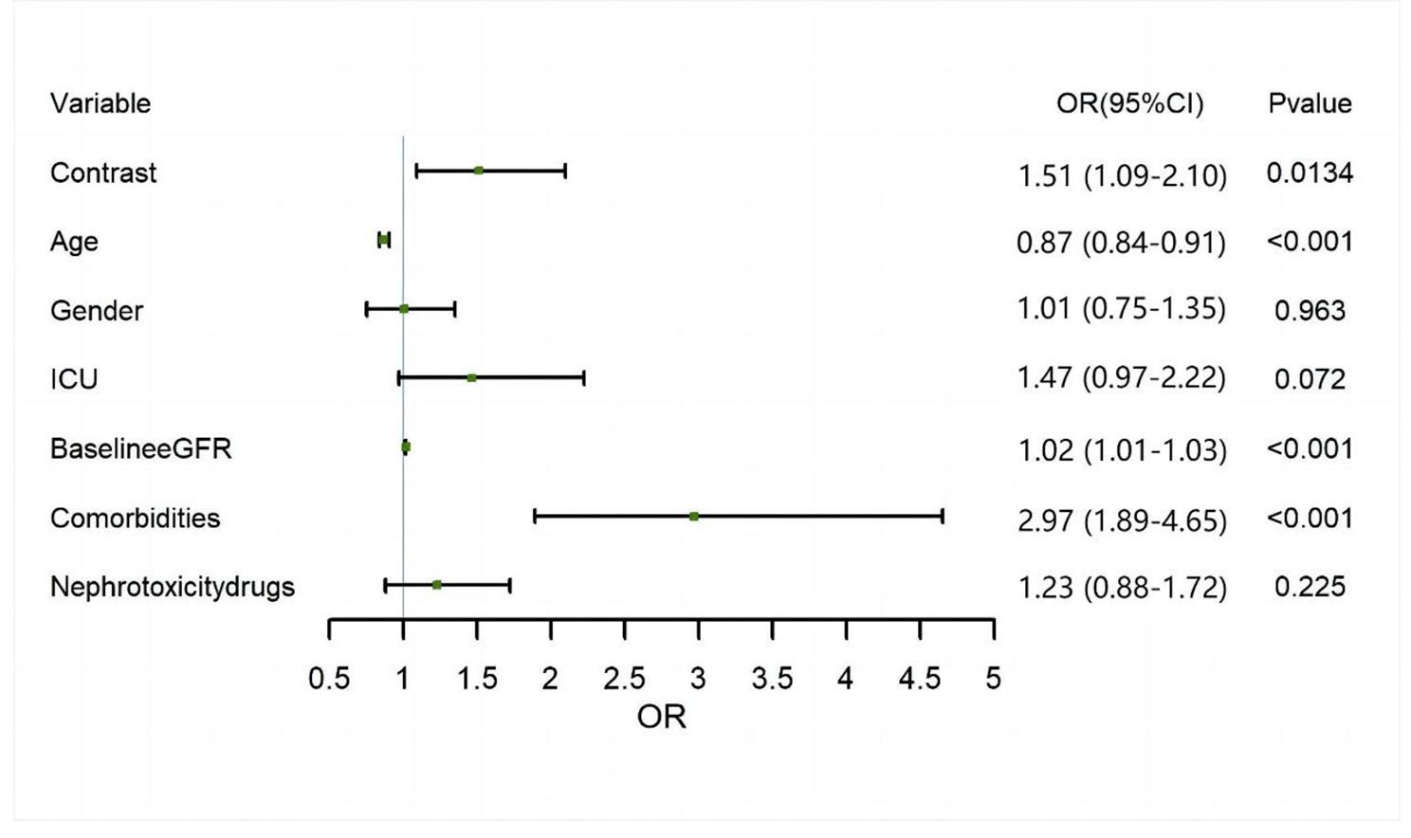
Factors associated with AKI according to multivariate logistic regression. ICU = Intensive Care Units

Finally, the multivariable logistic regression analysis was performed to identify other risk factors for AKI in pediatric patients via CT/MRI, such as age, sex (female), ICU admission, baseline eGFR, the number of comorbidities, and number of nephrotoxicity drugs. Odds ratios (ORs), 95% CIs, and P values among the different groups according to these models are presented in Fig. 2 and Supplemental Table 7. A P value less than 0.05 indicated Statistical significance. All the statistical analyses were performed by using SAS (version 9.4) and R (version 4.2).

## Results

From 2015 to 2020 at Beijing Children’s Hospital, 50,204 CT/MRI examinations were performed, accounting for 19.0% of the 264,131 hospital admissions. Among the 50,204 patients, 10,786 (21.5%) had available contrast-enhanced CT/MRI data. A total of 3061 pediatric patients were eligible for inclusion and exclusion; 2086 patients were exposed to contrast media, and the remaining 975 patients were not exposed to contrast media (Fig. 1). 1818 patients had CT examinations and 1243 patients had MRI examinations. The median age of the 3061 pediatric patients was 4.5 (IQR, 1.3–8.9) years, in which 57.5% of pediatric patients were males (*n* = 1760), and 10.2% of the pediatric patients admitted to ICU underwent CT/MRI examinations (*n* = 311). Detailed information about the baseline characteristics of participants receiving contrast media (exposure group) and not receiving contrast media (control group) is shown in Supplemental Results 1 and Supplemental Table 3.

According to the KDIGO guidelines, the overall incidence of AKI in 3061 pediatric patients was 7.1% (95% CI: 6.2-8.0%). As shown in Table 1 and Supplemental Fig. 3, the characteristics of the covariates between the exposure group and the control group were not comparable before applying IPTW, and the SMDs between the exposure group and the control group were greater than 0.1. The SMDs between the exposure group o and the control group were less than 0.1 after IPTW. Table 2 shows that the incidence of AKI in the exposure group and the control group was 7.4% (95% CI: 6.3-8.6%) and 6.5% (95% CI: 4.9-8.0%), respectively, and the aRR, based on log-binomial regression analyses, was 1.35 (95% CI: 1.10–1.65).

**Table 1:** Demographics and characteristics of participants

Baseline characteristics	Unweighted Cohort				Inverse Probability of Treatment Weighting Cohort		
	Exposure group (*n* = 2086)	Control group (*n* = 975)	SMD		Exposure group (*n* = 3066)	Control group (*n* = 3060)	SMD
Age (year), Mean ± SD	5.6 ± 4.4	5.1 ± 4.4	0.113		5.5 ± 4.4	5.5 ± 4.7	0.006
Male, n (%)	1181 (56.7)	579 (59.4)	0.056		1758 (57.3)	1756 (57.4)	0.001
ICU admission, n (%)	227 (10.9)	84 (8.6)	0.076		313.0 (10.2)	310 (10.1)	0.003
Baseline eGFR (ml/min/1.73m^2^), Mean ± SD	138.9 ± 57.9	141.5 ± 47.2	0.049		141.8 ± 76.4	141.3 ± 45.6	0.008
Comorbidities, n (%)							
Diabetes mellitus	51 (2.4)	64 (6.6)	0.200		116 (3.8)	114 (3.7)	0.004
Hypertension	18 (0.9)	21 (2.2)	0.106		36 (1.2)	36 (1.2)	0.002
Kidney disease	6 (0.3)	12 (1.2)	0.109		24 (0.8)	18 (0.6)	0.020
Sepsis	6 (0.3)	9 (0.9)	0.082		20 (0.7)	15 (0.5)	0.020
Cardiac failure	31 (1.5)	17 (1.7)	0.020		48 (1.6)	50 (1.6)	0.006
Respiratory failure	11 (0.5)	3 (0.3)	0.034		14 (0.5)	15 (0.5)	0.003
No. of nephrotoxicity drugs, n (%)			0.002				0.003
< 3	1613 (77.3)	753 (77.2)			2355 (76.8)	2346 (76.7)	
≥ 3	473 (22.7)	222 (22.8)			711 (23.2)	714 (23.3)	

Abbreviations: SD, Standard Difference; ICU, Intensive Care Units; eGFR, estimated Glomerular Filtration Rate; No., Number; SMD, Standardized Mean Difference.

**Table 2:** AKI Incidence and its 95% confidence intervals

	Total			Exposure group			Control group			Unweighted Cohort		IPTW Cohort
	*n*	Incidence, %		*n*	Incidence, %		*n*	Incidence, %		cRR (95% CI)		aRR (95% CI)
AKI in the full population	3061	7.1 (6.2, 8.0)		2086	7.4 (6.3, 8.6)		975	6.5 (4.9, 8.0)		1.15 (0.87, 1.54)		1.35 (1.10, 1.65)
AKI in patients underwent CT	1818	7.7 (6.5, 8.9)		1187	8.1 (6.5, 9.6)		631	7.0 (5.0, 9.0)		1.16 (0.83, 1.65)		1.39 (1.09, 1.78)
AKI in patients underwent MRI	1243	6.3 (4.9, 7.6)		899	6.6 (4.9, 8.2)		344	5.5 (3.1, 7.9)		1.19 (0.73, 2.02)		1.36 (0.96, 1.95)
AKI in patients < 2 years old	954	10.9 (8.9, 12.9)		618	11.7 (9.1, 14.2)		336	9.5 (6.4, 12.7)		1.22 (0.83, 1.84)		1.33 (0.99, 1.80)
AKI in patients ≥ 2 years old	2107	5.4 (4.4, 6.4)		1468	5.7 (4.5, 6.8)		639	4.9 (3.2, 6.5)		1.17 (0.79, 1.77)		1.38 (1.05, 1.82)
AKI in females	1301	7.6 (6.2, 9.1)		905	8.0 (6.2, 9.7)		396	6.8 (4.3, 9.3)		1.17 (0.77, 1.82)		1.41 (1.05, 1.89)
AKI in males	1760	6.8 (5.6, 7.9)		1181	7.0 (5.6, 8.5)		579	6.2 (4.3, 8.2)		1.13 (0.78, 1.67)		1.30 (0.99, 1.70)
AKI in sensitivity analysis*	3028	7.1 (6.2, 8.1)		2068	7.4 (6.3, 8.5)		960	6.6 (5.0, 8.1)		1.13 (0.85, 1.51)		1.32 (1.08, 1.61)

Abbreviations: AKI, Acute Kidney Injury; eGFR, estimated Glomerular Filtration Rate; IPTW, Inverse Probability of Treatment Weighting; cRR, crude Risk Ratio; aRR, adjusted Risk Ratio.

*In sensitivity analysis, patients with eGFR < 60 ml/min/1.73m^2^ were excluded.

In patients underwent CT, the risk of AKI in the exposure group was greater than that in the control group, and the aRR was 1.39 (95% CI: 1.09–1.78). However, it was not observed in patients underwent MRI (aRR: 1.36; 95% CI: 0.96–1.95). In the subgroup of pediatric patients aged ≥ 2 years, the risk of AKI in the exposure group was greater than that in the control group (aRR: 1.38; 95% CI: 1.05–1.82), which was different from the results of pediatric patients aged < 2 years (aRR: 1.33; 95% CI: 0.99–1.80). The increased risk attributed to exposure of contrast media is shown in females (aRR: 1.41, 95% CI: 1.05–1.89) instead of males (aRR: 1.30, 95% CI: 0.99–1.70). Moreover, the incidence of AKI in patients aged < 2 years old (10.9%, 95% CI: 8.9-12.9%) was higher than that in patients aged ≥ 2 years old (5.4%, 95% CI: 4.4-6.4%), but there was no difference between females (7.6%, 95% CI: 6.2-9.1%) and males (6.8%, 95% CI: 5.6-7.9%). Similarly, there was not significantly difference of the incidence of AKI between CT group and MRI group. Furthermore, the AKI incidence in the exposure group (7.4%, 95% CI: 6.3-8.5%) was greater than that in the control group (6.6%, 95% CI: 5.0-8.1%) when patients with a baseline eGFR less than 60 mL/min/1.73m^2^ were excluded, and aRR was 1.32 (95% CI: 1.08–1.61). The baseline characteristics of the participants in the exposure group and the control group in subgroup analysis and sensitivity analysis are shown in Supplemental Tables 4–6.

According to the results of multivariable logistic regression analysis, contrast media (OR: 1.51; 95% CI: 1.09–2.10), baseline eGFR (OR: 1.02; 95% CI: 1.01–1.03 for every 1 ml/min/1.73m^2^ increase in eGFR), and comorbidities (OR: 2.97; 95% CI: 1.89–4.65) were risk factors for AKI, while age (OR: 0.87; 95% CI: 0.84–0.91) was a protective factor against AKI (Fig. 2). Detailed results of the multivariate logistic regression analysis are shown in Supplemental Results 2 and Supplemental Table 7. Moreover, Supplemental Tables 8 and Supplemental Fig. 4 show that AKI did not occur within 48 h after exposure to contrast media.

## Discussion

Recently, a ten-year retrospective study revealed an increased risk of AKI in pediatric patients (with an eGFR ≥ 60 ml/min/1.73m^2^) exposed to intravenous contrast media compared with those without exposure to contrast media [11], which was different from the findings of previous studies regarding pediatric CA-AKI [13, 15]. Although the dosage of contrast media for pediatric patients has decreased, the risk of AKI related to contrast media exposure should not be ignored. According to the results of the present 6-year retrospective cohort study, the risk of AKI in the exposure group was greater than that in the control group after IPTW was used to reduce systematic differences in baseline characteristics among participants with or without exposure to contrast media, and similar results were observed in subgroup analyses and sensitivity analyses (all aRRs were more than 1). Therefore, the findings of the present study support the recommendations of the European Society of Urogenital Radiology nephropathy; i.e., before an intervention that encompasses a risk factor for CA-AKI, a baseline SCr level should be determined and a repeat SCr level should be performed 12 and 72 h after administration of contrast media in high-risk patients to determine the ratio of the benefits and harms of administering contrast media to children in the absence of known renal failure [10, 22].

On the other hand, the overall incidence of AKI was 7.1% before IPTW, and there was no statistically significant difference between the incidence of AKI in the exposure group and that in the control group (7.4% vs. 6.5%, *p* = 0.33). The 7.4% rate of CA-AKI in our research was greater than that previously reported in American children (1.4%, 2.4%, 3.3%) [11, 13, 15] due to the existence of different definitions of CA-AKI. Unlike previous research regarding pediatric CA-AKI, which identified the AKI based on an SCr within 48 h after imaging examinations [11, 13, 15], especially for pediatric CI-AKI [12, 16], our research selected the SCr tested within 7 days after CT/MRI for diagnosing AKI which is suitable for an updated definition of CA-AKI [2, 27]. Interestingly, the occurrence of AKI was not limited to 48 h after exposure to the contrast media, as shown in Supplemental Tables 8 and Supplemental Fig. 4. Since intravascular contrast media is excreted from the human body for at least 1–2 days and an obvious increase in SCr is not observed until at least 2 days after renal injury occurs [4, 28], it is feasible to extend the exposure period to seven days after contrast agent exposure to diagnose CA-AKI on the premise of excluing other causes of AKI, such as renal perfusion pressure decreasing induced by acute hemorrhage, renal vascular or interstitial lesions, or urinary tract obstruction.

Most researchers agree that the contrast agents used for CT and MRI have different risk profiles for kidney injury. Therefore, the subgroup analysis by the type of imaging examinations (CT/MRI) was performed and the results clarified that the increased risk attributed to exposure iodine-based contrast medium in CT instead of gadolinium-based contrast media, which was consistent with previous findings that gadolinium-based contrast media generally leads to subclinical kidney damage without any significant modification of SCr [6, 7]. The aRR for the risk of AKI after exposure to gadolinium-based contrast media was more than 1, although 95% CI included 1, which remand us the potential kidney injury of gadolinium-based contrast did not be ignored. In addition, the development pattern of renal function (commonly represented by the GFR) in children ranged from 0 to 2 years old, gradually developed with age and approached adult levels until 2 years of age (GFR > 100 mL/min/1.73 m^2^) [3]. According to our subgroup analysis (stratified by age), the median baseline eGFR in the group aged < 2 years (114.6 mL/min/1.73 m^2^) was lower than that in the group aged ≥ 2 years (140.9 mL/min/1.73 m^2^). Similarly, the incidence of AKI in patients aged < 2 years was greater than that in patients aged ≥ 2 years, which indicated that younger children have a greater risk of AKI after exposure to contrast media. The risk of AKI in patients aged < 2 years whose renal function was relatively undeveloped increased compared with that in patients aged ≥ 2 years after IPTW. However, the risk of AKI due to contrast media in pediatric patients was different from that in adult studies in which there was no evidence of an increased risk of AKI due to contrast media [29, 30]. Therefore, in addition to the baseline eGFR, there may be additional factors involved in the occurrence of AKI in pediatric patients.

According to the sensitivity analyses, after patients with an eGFR < 60 ml/min/1.73m^2^ were excluded, the results still showed a similar increase risk of AKI due to exposure to contrast media. Calle-Toro et al. also showed an increased risk of AKI with exposure to contrast agent only in pediatric patients with an eGFR ≥ 60 mL/min/1.73m^2^, without considering comorbidities or nephrotoxicity drugs as confounders. McDonald et al. reported that the risk of AKI (stage 2 AKI) in the exposure group was 2.00 times greater than that in the control group following propensity score adjustment, but its 95% CI included 1. This may be limited by the small sample size (*n* = 710) [15]. Gilligan et al. reported that the risk of AKI in the exposure group was 0.91 times greater than that in the control group with abdominal US using 1:1 propensity score matching (*n* = 1850); however, its 95% CI included 1, in which the small sample size and low incidence of postcontrast AKI limited the ability to detect an effect of contrast agent administration on the outcome [13]. Unlike the above PS matching, which led to the loss of cases [31], the IPTW used in our research fully utilized the participants included in this cohort.

According to the results of multivariable logistic regression analysis, contrast media and comorbidities were risk factors for AKI, while age was a protective factor against AKI; these findings were similar to the findings of pediatric research regarding CA-AKI [11, 13, 15]. Despite the OR being close to 1, the baseline eGFR became a risk factor for AKI in pediatric patients, which was not consistent with clinical experience. The reason for the above counterintuitive results was that SCr was negatively correlated with eGFR according to the FAS equation for calculating eGFR; the higher the baseline eGFR was, the more easily SCr after imaging examinations increased 1.5 folds to diagnose AKI.

However, our research has several limitations. First, a large number of children who underwent CT/MRI but had insufficient pre- or post-CT/MRI SCr data within a specific time window were excluded from our research, which may have affected our findings. Second, this cohort did not include sufficient children with a baseline eGFR < 60 mL/min/1.73 m^2^, so an analysis stratified by baseline eGFR was not performed. Third, in contrast-enhanced CT/MRI, the exposure dose and type of contrast media used differed among anatomic sections, but the dose‒response relationship between exposure to contrast media and AKI was not considered due to a lack of detailed information. Fourth, information bias and admission rate bias cannot be ignored in this single-center retrospective cohort study. Beijing Children’s Hospital is a tertiary general children’s hospital with the highest comprehensive strength in China, so the admission rates of rare diseases in Beijing Children’s Hospital are significantly higher than other children’s hospital in China, for example the frequency of type 1 diabetes mellitus appears high for children in this study. The routine electronic medical record data did not collect the detailed information on some clinical parameters such as blood pressure, or use of inotropic support etc. Therefore, unmeasured confounders may remain, although PS and IPTW analyses were performed to reduce the SMD of confounders at baseline among the groups. Meanwhile, PS analysis can never fully adjust confounders due to the absence of clear indications for enhanced imaging studies. Fifth, the children in the cohort did not receive routine hydration therapy during the imaging study. If hydrated, a subset of the children could potentially be prevented from developing AKI. Thus, the estimated risk of AKI presented in this study might be overestimated. Sixth, although the patients with other causes of AKI (such as renal perfusion pressure decreasing induced by acute hemorrhage, renal vascular or interstitial lesions, or urinary tract obstruction) were excluded in this study, it is complex to diagnose the etiology of AKI. It was unavoidable that some possible causes of AKI, happened during these 7 days, were not taken into consideration in this study, which may affected the association between contrast and the risk of AKI. Finally, neonates younger than 28 days were not included in our research due to the lack of normal reference intervals of SCr [32].

## Conclusions

Therefore, the evidence based on the present study reminded us that the increased risk for AKI in hospitalized children induced by intravascular contrast should not be ignored, especially for patients aged < 2 years old or exposed to iodine-based contrast, but it is necessary to further validate the association between exposure to contrast media and AKI in the prospective study with a large sample.

### Electronic supplementary material

Below is the link to the electronic supplementary material.

Supplementary Material 1

## Data Availability

The datasets generated and/or analyzed during the current study are not publicly available due the data in this research were collected from electronic medical records at Beijing Children’s Hospital, and it is our duty to protect pediatric patients’ privacy; moreover, the data are available from the corresponding author upon reasonable request.

## Abbreviations

CA-AKI: Contrast-Associated Acute Kidney Injury
CT: Computed Tomography
MRI: Magnetic Resonance Imaging
CI-AKI: Contrast-Induced Acute Kidney Injury
AKI: Acute Kidney Injury
SCr: Serum Creatinine
KDIGO: Kidney Disease: Improving Global Outcomes
eGFR: estimated Glomerular Filtration Rate
ICU: Intensive Care Nnits
OR: Odds Ratios
RR: Risk Ratios
cRR: crude Risk Ratio
aRR: adjusted Risk Ratio
SD: Standard Difference
IQR: InterQuartile Range
SMD: Standardized Mean Difference
IPTW: Inverse Probability of Treatment Weighting
PS: Propensity Score

## References

[1] Kang C, Han SH, Park JS, Choi DE (2023). Risk factors for post-contrast acute kidney injury in patients sequentially administered iodine- and gadolinium-based contrast media on the same visit to the emergency department: a retrospective study. Kidney Res Clin Pract.

[2] Mehran R, Dangas GD, Weisbord SD (2019). Contrast-Associated Acute kidney Injury. N Engl J Med.

[3] Schwartz GJ, Work DF (2009). Measurement and estimation of GFR in children and adolescents. Clin J Am Soc Nephrol.

[4] Fahling M, Seeliger E, Patzak A, Persson PB (2017). Understanding and preventing contrast-induced acute kidney injury. Nat Rev Nephrol.

[5] Rudnick MR, Wahba IM, Leonberg-Yoo AK, Miskulin D, Litt HI (2021). Risks and options with gadolinium-based contrast agents in patients with CKD: a review. Am J Kidney Dis.

[6] Elmståhl B, Leander P, Grant D, Doughty RW, Chai CM, Björk J, Almén T, Nyman U (2007). Histomorphological changes after renal X-ray arteriography using iodine and gadolinium contrast media in an ischemic porcine model. Acta Radiol.

[7] Martino F, Amici G, Godi I, Baretta M, Biasi C, Carta M, Corradi V, De Cal M, Knust M, Tamayod C (2021). Gadolinium-based contrast media exposure and the possible risk of subclinical kidney damage: a pilot study. Int Urol Nephrol.

[8] Finn WF (2006). The clinical and renal consequences of contrast-induced nephropathy. Nephrol Dialysis Transplantation.

[9] McCullough PA, Choi JP, Feghali GA, Schussler JM, Stoler RM, Vallabahn RC, Mehta A (2016). Contrast-Induced Acute kidney Injury. J Am Coll Cardiol.

[10] Stacul F, van der Molen AJ, Reimer P, Webb JA, Thomsen HS, Morcos SK, Almen T, Aspelin P, Bellin MF, Clement O (2011). Contrast induced nephropathy: updated ESUR Contrast Media Safety Committee guidelines. Eur Radiol.

[11] Calle-Toro J, Viteri B, Ballester L, Garcia-Perdomo HA, White A, Pradhan M, Otero HJ (2023). Risk of Acute kidney Injury following contrast-enhanced CT in a cohort of 10 407 children and adolescents. Radiology.

[12] Agarwal Y, Rameshkumar R, Krishnamurthy S, Senthilkumar GP (2021). Incidence, risk factors, the role of plasma NGAL and outcome of contrast-Induced Acute kidney Injury in critically Ill Children. Indian J Pediatr.

[13] Gilligan LA, Davenport MS, Trout AT, Su W, Zhang B, Goldstein SL, Dillman JR (2020). Risk of Acute kidney Injury following contrast-enhanced CT in hospitalized Pediatric patients: a propensity score analysis. Radiology.

[14] Tkaczyk M, Tomczyk D, Jander A, Góreczny S, Moszura T, Dryżek P, Krajewski W, Głowacka E, Wosiak A (2018). Glomerular filtration decrease after diagnostic cardiac catheterisation in children with congenital cardiac malformation – the role of serum creatinine, cystatin C, neutrophil gelatinase and urine output monitoring. Adv Interventional Cardiol.

[15] McDonald JS, McDonald RJ, Tran CL, Kolbe AB, Williamson EE, Kallmes DF (2018). Postcontrast Acute kidney Injury in Pediatric patients: a Cohort Study. Am J Kidney Dis.

[16] Cantais A, Hammouda Z, Mory O, Patural H, Stephan JL, Gulyaeva L, Darmon M (2016). Incidence of contrast-induced acute kidney injury in a pediatric setting: a cohort study. Pediatr Nephrol.

[17] Hwang YJ, Hyun MC, Choi BS, Chun SY, Cho MH (2014). Acute kidney injury after using contrast during cardiac catheterization in children with heart disease. J Korean Med Sci.

[18] Soomro QH, Anand ST, Weisbord SD, Gallagher MP, Ferguson RE, Palevsky PM, Bhatt DL, Parikh CR, Kaufman JS (2022). The relationship between rate and Volume of Intravenous Fluid Administration and kidney outcomes after Angiography. Clin J Am Soc Nephrol.

[19] Aycock RD, Westafer LM, Boxen JL, Majlesi N, Schoenfeld EM, Bannuru RR (2018). Acute kidney Injury after computed tomography: a Meta-analysis. Ann Emerg Med.

[20] McDonald JS, Leake CB, McDonald RJ, Gulati R, Katzberg RW, Williamson EE, Kallmes DF (2016). Acute kidney Injury after Intravenous Versus Intra-arterial Contrast Material Administration in a paired cohort. Invest Radiol.

[21] Song W, Yan R, Peng M, Jiang H, Li G, Cao S, Jiang Y, Guo Z, Chen D, Yang H (2022). Age and sex specific reference intervals of 13 hematological analytes in Chinese children and adolescents aged from 28 days up to 20 years: the PRINCE study. Clin Chem Lab Med.

[22] Fliser D, Laville M, Covic A, Fouque D, Vanholder R, Juillard L, Van Biesen W, Ad-hoc working group of E (2012). A European renal best practice (ERBP) position statement on the kidney disease improving global outcomes (KDIGO) clinical practice guidelines on acute kidney injury: part 1: definitions, conservative management and contrast-induced nephropathy. Nephrol Dial Transpl.

[23] Pottel H, Hoste L, Dubourg L, Ebert N, Schaeffner E, Eriksen BO, Melsom T, Lamb EJ, Rule AD, Turner ST (2016). An estimated glomerular filtration rate equation for the full age spectrum. Nephrol Dial Transpl.

[24] Haukoos JS, Lewis RJ. The Propensity Score. *Jama* 2015, 314(15). 10.1001/jama.2015.13480 PMC4866501 26501539

[25] Bettega F, Leyrat C, Tamisier R, Mendelson M, Grillet Y, Sapène M, Bonsignore MR, Pépin JL, Kattan MW, Bailly S (2022). Application of inverse-probability-of-treatment weighting to Estimate the Effect of Daytime Sleepiness in patients with obstructive sleep apnea. Annals Am Thorac Soc.

[26] Austin PC, Stuart EA (2015). Moving towards best practice when using inverse probability of treatment weighting (IPTW) using the propensity score to estimate causal treatment effects in observational studies. Stat Med.

[27] Mandurino-Mirizzi A, Munafò A, Crimi G. Contrast-Associated Acute kidney Injury. J Clin Med 2022, 11(8). 10.3390/jcm11082167 PMC9027950 35456260

[28] Davies J, Siebenhandl-Wolff P, Tranquart F, Jones P, Evans P (2022). Gadolinium: pharmacokinetics and toxicity in humans and laboratory animals following contrast agent administration. Arch Toxicol.

[29] McDonald JS, McDonald RJ, Carter RE, Katzberg RW, Kallmes DF, Williamson EE (2014). Risk of intravenous contrast material-mediated acute kidney injury: a propensity score-matched study stratified by baseline-estimated glomerular filtration rate. Radiology.

[30] McDonald JS, McDonald RJ, Comin J, Williamson EE, Katzberg RW, Murad MH, Kallmes DF (2013). Frequency of acute kidney injury following intravenous contrast medium administration: a systematic review and meta-analysis. Radiology.

[31] Kane LT, Fang T, Galetta MS, Goyal DKC, Nicholson KJ, Kepler CK, Vaccaro AR, Schroeder GD (2020). Propensity score matching: a statistical method. Clin Spine Surg.

[32] Peng X, Peng Y, Zhang C, Zhao M, Yang H, Cao S, Li G, Jiang Y, Guo Z, Chen D (2022). Reference intervals of 14 biochemical markers for children and adolescence in China: the PRINCE study. Clin Chem Lab Med.

