# Supplementary material for: Risk of acute kidney injury following contrast-enhanced CT or MRI in a cohort of 3061 hospitalized children in China
Source: BMC Pediatr. 2024 Jun 19;24:400. doi: 10.1186/s12887-024-04875-z (PMC11186257; doi:10.1186/s12887-024-04875-z)
Supplement: Supplementary file 1 — Supplementary Material 1 [file 12887_2024_4875_MOESM1_ESM.docx]

**Risk of Acute Kidney Injury Following Contrast-enhanced CT or MRI in a Cohort of 3061 Hospitalized Children in China**

**-Supplementary Materials**

Supplemental Method 1 Contrast material administration and oral hydration administration in CT/MRI

Supplemental Results 1 The baseline characteristics of exposure group of contrast media and control group

Supplemental Results 2 Multivariate logistic regression analysis

Supplemental Table 1 Dosages of iodine contrast media.

Supplemental Table 2 The detailed list of nephrotoxicity drugs used in our study.

Supplemental Table 3 Characteristics of 3061 pediatric patients treated with or without contrast media.

Supplemental Table 4 Patients characteristics in subgroup by the type of imaging examinations.

Supplemental Table 5 Patients characteristics in subgroup by age

Supplemental Table 6 Patients characteristics in subgroup by eGFR

Supplemental Table 7 Results of multivariate logistic regression

Supplemental Table 8 The distribution of children with a serum creatinine and diagnosed by AKI in 1-7 days after contrast exposure.

Supplemental Figure 1 Distribution of propensity scores in exposure group and control group.

Supplemental Figure 2 Standardized mean differences (SMDs) of different variables between the exposure group and the control group in unmatched population and inverse probability of treatment weighting (IPTW) cohort.

Supplemental Figure 3 Distribution of weights generated by the inverse probability of treatment weighting.

Supplemental Figure 4 The distribution of children diagnosed with AKI in 1-7 days after contrast agent exposure.

**Supplemental Method 1 Contrast material administration and oral hydration administration in CT/MRI examination**

Enhanced CT protocol: All contrast-enhanced CT examinations performed in patients included in this study had been performed with a low-osmolality iodinated contrast media, such as iohexol (GE Health care America), iopamidol, ioversol, iopromide or an iso-osmotic iodine contrast media, iodixanol. CT examinations were classified by anatomic section as follows: a) head, including the brain, orbits, face, temporal bones, and sinuses; b) full body, chest, abdominal, pelvic, and lumbar spine; c) extremities and arthrosis; d) Heart and coronary artery. Iodinated contrast agent was administered intravenously based on patient’s weight (**Table 1**) using a single-head power inject (except heart and coronary artery examination) or double-head power inject (heart and coronary artery examination only) at injection rates of 0.4–5.0 ml/s, the maximum dose was 70ml.

Enhanced MR protocol: The contrast media in MRI examinations was gadolinium-based contrast agent included gadopentetate dimeglumine (Magnevist) and gadoterate meglumine (Dotarem). MRI examinations were classified by anatomic section as follows: a) head, including the brain, orbits, face, and sinuses; b) pituitary gland; c) full body, including chest, abdominal, and pelvic studies; d) extremities and arthrosis(3). Gadolinium-based contrast agent was administered intravenously based on patient’s weight (0.2ml/kg for head and body examination and 0.1ml/kg for pituitary gland examination) using a double-head power inject at injection rates of 0.8-1.5 ml/s. The maximum dose was 20ml for head and body examination and 10ml for pituitary gland examination.

The protocol of hydration administration in CT/MRI examination: All pediatric patients with intravenous contrast-enhanced CT/MRI are recommended to comply with the oral hydration administration before the intravascular injection of contrast material, i.e., they are allowed to drink large amounts of water or to shorten the duration of water deprivation. Iintravenous hydration administration was not performed in our hospital setting due to the young age, light weight, and poor compliance of invasive procedures.

The change of CT/MRI protocols in Beijing Children’s Hospital during 2015-2020: According to the advanced experience of low contrast agent research in children, the total amount of contrast agent in contrast-enhanced CT/MRI in children was adjusted in our hospital. In general, the amount of contrast agent used in contrast-enhanced CT in children was gradually reduced, and the degree of reduction was gradually different in different age groups. The most obvious dose of changes for iodine-based contrast medium were observed in 2014 and 2020. The same amount of gadolinium-based contrast material was given during 2015-2020 in Beijing Children’s Hospital.

**Supplemental Results 1 The baseline characteristics of exposure group of contrast media and control group**

In exposure group of contrast media, the median age was 4.8 (IQR, 1.4-9.2) years, and 56.7% were male (n=1181); in control group, the median age was 3.9 (IQR, 1.1–8.2) years, and 59.4% were male (n=579). In total 3061 pediatric patients, the exposure group of contrast media were relatively older and had lower eGFR and fewer comorbidities (such as diabetes mellitus, hypertension, kidney disease, and sepsis) compared with the control group (P <0.05) (**Table 3**). The raw incidence rate of AKI (defined by KDIGO guideline) was 7.1% for the entire sample (218 of 3061); it was 7.4% (155 of 2086) in the exposure group of contrast media and 6.5% (63 of 975) in control group (P = 0.33) (**Table 3**).

Subgroup analysis with stratification by the type of imaging examinations indicated that the exposure group of contrast media had no significantly higher incidence of AKI than control group in both patients underwent CT (8.1% versus 7.0%, P = 0.40) and patients with MRI (6.6% versus 5.5%, P = 0.50) (**Table 4**). Substantial differences of in age, ICU admission, baseline SCr, and comorbidities were seen between exposure group of contrast media and control group (p<0.05) (**Table 4**).

Subgroup analysis with stratification by age revealed that patients aged <2 years had higher risk for AKI, with an overall incidence of 10.9% (104 of 954) and 5.4% (114 of 2107) in patients aged ≥2 years (P < 0.01). When compared with control group, the exposure group of contrast media had no significantly higher incidence of AKI in both patients aged <2 years old (11.7% versus 9.5%, P = 0.53) and patients aged ≥2 years old (5.7% versus 4.9%, P = 0.31) (**Table 5**). Meanwhile, the baseline median eGFRs in patients aged <2 years old were lower than that in patients aged ≥2 years old (114.6 versus 140.9 mL/min/1.73 m^2^, P < 0.01). Substantial differences in age, ICU admission, baseline eGFRs, and comorbidities were seen between exposure group of contrast media and control group (p<0.05) (**Table 5**).

In sensitivity analysis when the patients with baseline eGFR less than 60 mL/min/1.73 m^2^ were excluded (n=3028), the median age was 4.5 (IQR, 1.3–8.9) years, and 57.3% were male (n=1735). The median age in exposure group of contrast media was more than that in control group (4.9 vs. 3.9 years, p<0.01). The exposure group of contrast media had lower eGFR and fewer comorbidities (especially diabetes mellitus, hypertension, kidney disease, and sepsis) compared with the control group (P <0.05) (**Table 6**). These patients had an AKI incidence of 7.4% (153 of 2068) when receiving contrast media compared with 6.6% (63 of 960) when studies were performed without contrast media (P=0.41) (**Table 6**). For the propensity-matched analysis, the AKI incidence in exposure group of contrast media and control group were 8.5% (78 of 922) and 6.2% (57 of 922), respectively (P= 0.06). In the propensity-matched group, there was a balance in age, gender, comorbidities, and the number of nephrotoxicity drugs, but a worse balance in baseline eGFR and ICU admission between exposure group of contrast media and control group (**Figure 1**).

**Supplemental Results 2 Multivariate logistic regression analysis**

In the full-population model (OR=1.51; 95% CI: 1.09, 2.10; p=0.01), the model for patients underwent CT (OR=1.44; 95% CI: 0.96, 2.17; p=0.08), the model for patients aged <2 years old (OR=1.89; 95% CI: 1.16, 3.08; p=0.01), the model for patients after patients with eGFR <60 ml/min/1.73 m^2^ were excluded (OR=1.49; 95% CI: 1.07, 2.07; p=0.02), and the propensity-matched model (OR=1.55; 95% CI: 1.06, 2.27; p=0.02), exposure of contrast media was associated with AKI after adjustment for age, gender, ICU admission, baseline eGFR, number of comorbidities, and number of nephrotoxicity drugs. In patients aged ≥2 years old (OR=1.29; 95% CI: 0.82, 2.02; p=0.27) and patients underwent MRI OR=1.39; 95% CI: 0.78, 2.48; p=0.26), exposure of contrast media was not associated with AKI (**Table 7, Figure 2**).

In terms of other model covariates, risk models of the full population demonstrated age differences, with older patients having a protective effect against AKI after controlling for the other variables included in the model (OR=0.87; 95% CI: 0.84, 0.91; p<0.01), which was equal to the results of the models in patients with eGFR ≥60 mL/min/1.73 m^2^ , and similar with the results of the models in the subgroup analyses (For patients aged <2 years old: OR=0.69; 95% CI: 0.41, 0.99; p<0.05; for patients aged ≥2 years old: OR=0.94; 95% CI: 0.89, 0.99; p=0.03; for patients underwent CT: OR=0.83; 95% CI: 0.79, 0.88; p<0.01; for patients underwent MRI: OR=0.92; 95% CI: 0.87, 0.98; p<0.01) and in the propensity-matched group (OR=0.83; 95% CI: 0.78, 0.88; p<0.01) (**Table 7**). Besides, the number of comorbidities (diabetes mellitus, hypertension, kidney disease, sepsis, cardiac failure, and respiratory failure) was a risk factor for AKI after adjustment for the other variables in full population model (OR=2.97; 95% CI: 1.89, 4.65; p<0.01), the model for patients underwent CT (OR=4.02; 95% CI: 2.38, 6.82; p<0.01), the model for patients aged ≥2 years old (OR=3.67; 95% CI: 2.11, 6.36; p<0.01), the model for patients with eGFR ≥60 mL/min/1.73 m^2^ (OR=3.03; 95% CI: 1.93, 4.76; p<0.01), and the propensity-matched model (OR=4.06; 95% CI: 1.93,4.76; p<0.01) (**Table 7**). However, the baseline eGFR became a risk factor after controlling for the other variables in all analyses (OR=1.02; 95% CI: 1.01, 1.03; p<0.01) (**Table 7**).

**Supplemental Table 1 Dosages of iodine contrast media.**

| Body weight/ kg | Dosage of iodine contrast media (ml/kg) | | | |
| --- | --- | --- | --- | --- |
|  | head | full body | extremities and arthrosis | heart and coronary artery |
| <10 | 1.6-2.2 | 1.5-2.1 | 2.0 | 1.6-1.8 |
| 10.1-20 | 0.8-2.0 | 1.3-1.9 | 2.0 | 1.2-1.4 |
| 20.1-35 | 0.8-1.5 | 1.0-1.5 | 1.5-1.8 | 1.2 |
| 35.1-50 | 0.8-1.0 | 1.0-1.2 | 1.4-1.6 | 1.0 |
| >50 | 50.0 | 1.0-1.2 | 70.0 | 1.0 |

**Supplemental Table 2 The detailed list of nephrotoxicity drugs used in our study.**

| Drug name | The types of nephrotoxicity drugs |
| --- | --- |
| Amikacin | Aminoglycoside antibiotics |
| Tobramycin | Aminoglycoside antibiotics |
| Mannitol | Diuretics |
| Furosemide | Diuretics |
| Spironolactone | Diuretics |
| Hydrochlorothiazide | Diuretics |
| Aspirin | NSAIDs |
| Ibuprofen | NSAIDs |
| Acetaminophen | NSAIDs |
| Celecoxib | NSAIDs |
| Diclofenac diethylamine | NSAIDs |
| Diclofenac sodium | NSAIDs |
| Diclofenac | NSAIDs |
| Esomeprazole magnesium | PPIs |
| Esomeprazole magnesium | PPIs |
| Omeprazole magnesium | PPIs |
| Omeprazole | PPIs |
| Omeprazole Sodium | PPIs |
| Vancomycin | Vancomycin |

Abbreviations: NSAIDs, Nonsteroidal anti-inflammatory drugs; PPIs, Proton Pump Inhibitors.

**Supplemental Table 3 Characteristics of 3061 pediatric patientstreated with or without contrast media.**

| Characteristic | Total | Exposure group | Control group | P |
| --- | --- | --- | --- | --- |
| No. of patients | 3061 | 2086 (68.1) | 975 (31.9) |  |
| Age (year), M (IQR) | 4.5 (1.3,8.9) | 4.8 (1.4, 9.2) | 3.9 (1.1,8.2) | <0.01 |
| Male, n (%) | 1760 (57.5) | 1181 (56.7) | 579 (59.4) | 0.15 |
| ICU admission | 311 (10.2) | 227 (10.9) | 84 (8.6) | 0.05 |
| Baseline renal function, M (IQR) |  |  |  |  |
| Baseline eGFR (ml/min/1.73m^2^) | 132.4 (110.3,158.7) | 131.5 (109.7,157.2) | 135.2 (111.9,162.6) | 0.02 |
| Baseline SCr (μmol/L) | 26.5 (20.6,35.7) | 27.2 (21.3,36.1) | 25.2 (29.3,34.7) | <0.01 |
| Comorbidities, n (%) |  |  |  |  |
| Diabetes mellitus | 115 (3.8) | 51 (2.4) | 64 (6.6) | <0.01 |
| Hypertension | 39 (1.3) | 18 (0.9) | 21 (2.2) | <0.01 |
| Kidney disease | 18 (0.6) | 6 (0.3) | 12 (1.2) | <0.01 |
| Sepsis | 15 (0.5) | 6 (0.3) | 9 (0.9) | 0.02 |
| Cardiac failure | 48 (1.6) | 31 (1.5) | 17 (1.7) | 0.59 |
| Respiratory failure | 14 (0.5) | 11 (0.5) | 3 (0.3) | 0.70 |
| No. of nephrotoxicity drugs, n (%) |  |  |  |  |
| ≥3 | 695 (22.7) | 473 (22.7) | 222 (22.8) | 0.95 |
| Renal function after contrast exposure, M (IQR) |  |  |  |  |
| eGFR (ml/min/1.73m^2^) | 128.9 (107.9,152.6) | 127.9 (107.2,151.5) | 131.4 (109.1,155.9) | 0.02 |
| SCr examination time(days) | 3.3 (1.4,5.1) | 3.4 (1.6,5.3) | 2.8 (0.9,4.8) | <0.01 |
| SCr (μmol/L) | 25.6 (20.7,33.2) | 28.7 (22.3,37.2) | 26.4 (20.0,34.6) | <0.01 |
| AKI (KDIGO), n (%) | 218 (7.1) | 155 (7.4) | 63 (6.5) | 0.33 |
| AKI stage (KDIGO), n (%) |  |  |  |  |
| Stage 1 | 119 (3.9) | 82 (3.9) | 37 (3.8) | 0.01 |
| Stage 2 | 49 (1.6) | 29 (1.4) | 20 (2.1) |  |
| Stage 3 | 50 (1.6) | 44 (2.1) | 6 (0.6) |  |

Abbreviations: No., Number; ICU, Intensive Care Unit; eGFR, estimated Glomerular Filtration Rate; SCr, Serum Creatine; AKI, Acute Kidney Injury; pRIFLE, pediatric Risk, Injury, Failure, Loss, End-stage renal disease; KDIGO, Kidney Disease: Improving Global Outcomes.

**Supplemental Table 4 Patients characteristics in subgroup by the type of imaging examinations.**

| Characteristic | Patients underwent CT (N=1818) | | |  | Patients underwent MRI (n=1243) | | |
| --- | --- | --- | --- | --- | --- | --- | --- |
|  | Exposure group | Control group | P |  | Exposure group | Control group | P |
| No. of patients | 1187 | 631 |  |  | 899 | 344 |  |
| Age (year), M (IQR) | 4.8 (1.6, 9.1) | 4.3 (1.4, 8.5) | 0.24 |  | 9.3 (4.9, 12.2) | 2.8 (0.9, 7.8) | \| <0.01 \| \| --- \| |
| Male, n (%) | 686 (57.8) | 387 (61.3) | 0.14 |  | 495 (55.1) | 192 (55.8) | 0.81 |
| ICU admission, n (%) | 152 (12.8) | 36 (5.7) | <0.01 |  | 75 (8.3) | 48 (14.0) | <0.01 |
| Baseline renal function, M (IQR) |  |  |  |  |  |  |  |
| Baseline eGFR (ml/min/1.73m^2^) | 132.9 (108.7, 160.3) | 136.9 (114.6, 161.5) | 0.03 |  | 130.3 (110.8, 152.5) | 130.1 (108.8, 163.2) | 0.68 |
| Baseline SCr (μmol/L) | 26.9 (21.7-35.7) | 25.8 (19.6-35.5) | <0.01 |  | 27.5 (20.6, 36.6) | 24.3 (19.2, 32.4) | \| <0.01 \| \| --- \| |
| Comorbidities, n (%) |  |  |  |  |  |  |  |
| Diabetes mellitus | 30 (2.5) | 23 (3.7) | 0.18 |  | 21 (2.4) | 41 (11.9) | <0.01 |
| Hypertension | 10 (0.8) | 5 (0.8) | 0.91 |  | 8 (0.9) | 16 (4.7) | <0.01 |
| Kidney disease | 2 (0.2) | 7 (1.1) | <0.01 |  | 4 (0.4) | 5 (1.5) | 0.13 |
| Sepsis | 3 (0.3) | 4 (0.6) | 0.21 |  | 3 (0.3) | 5 (1.5) | 0.04 |
| Cardiac failure | 31 (2.6) | 16 (2.5) | 0.92 |  | 0 (0.0) | 1 (0.3) | 0.28 |
| Respiratory failure | 10 (0.8) | 1 (0.2) | 0.07 |  | 1(0.1) | 2 (0.6) | 0.19 |
| No. of nephrotoxicity drugs, n (%) |  |  |  |  |  |  |  |
| ≥3 | 209 (17.6) | 142 (22.5) | 0.01 |  | 264 (29.4) | 80 (23.3) | 0.03 |
| Renal function after contrast exposure, M (IQR) |  |  |  |  |  |  |  |
| eGFR (ml/min/1.73㎡) | 127.6 (104.7, 152.7) | 134.3 (111.5, 158.4) | <0.01 |  | 128.4 (109.2, 150.1) | 126.1 (104.7, 149.9) | 0.45 |
| SCr examination time after contrast exposure (days) | 3.6 (1.6, 5.4) | 2.6 (0.8, 4.8) | <0.01 |  | 3.3 (1.6, 5.3) | 3.1 (1.5, 4.8) | 0.20 |
| SCr (μmol/L) | 29.0 (22.6, 37.1) | 26.9 (20.2, 35.0) | <0.01 |  | 28.5 (21.2, 37.3) | 25.7 (19.7, 34.3) | <0.01 |
| AKI (KDIGO), n (%) | 96 (8.1) | 44 (7.0) | 0.40 |  | 59 (6.6) | 19 (5.5) | 0.50 |
| AKI stage (KDIGO), n (%) |  |  |  |  |  |  |  |
| Stage 1 | 52 (4.4) | 26 (4.1) | 0.64 |  | 30 (3.3) | 11 (3.2) | <0.01 |
| Stage 2 | 24 (2.0) | 12 (1.9) |  |  | 5 (0.6) | 8 (2.3) |  |
| Stage 3 | 20 (1.7) | 6 (1.0) |  |  | 24 (2.6) | 0 (0.0) |  |

Abbreviations: No., Number; ICU, Intensive Care Unit; eGFR, estimated Glomerular Filtration Rate; SCr, Serum Creatine; AKI, Acute Kidney Injury; KDIGO, Kidney Disease: Improving Global Outcomes.

**Supplemental Table 5 Patients characteristics in subgroup by age**

| Characteristic | Age<2 years old | | |  | Age≥2 years old | | |
| --- | --- | --- | --- | --- | --- | --- | --- |
|  | Exposure group | Control group | P |  | Exposure group | Control group | P |
| No. of patients | 618 | 336 |  |  | 1468 | 639 |  |
| Age (year), M (IQR) | 0.6 (0.3, 1.1) | 0.8 (0.4, 1.2) | 0.02 |  | 7.2 (4.4, 10.6) | 6.9 (3.9, 10.3) | 0.04 |
| Male, n (%) | 364 (58.9) | 204 (60.7) | 0.59 |  | 817 (55.7) | 375 (58.7) | 0.20 |
| ICU admission, n (%) | 116 (18.8) | 41 (12.2) | <0.01 |  | 111 (7.6) | 43 (6.7) | 0.50 |
| Baseline renal function, M (IQR) |  |  |  |  |  |  |  |
| Baseline eGFR (ml/min/1.73m^2^) | 112.3 (95.2, 133.7) | 121.2 (100.0, 148.1) | <0.01 |  | 139.6 (117.5, 164.5) | 142.9 (117.4, 167.0) | 0.32 |
| Baseline SCr (μmol/L) | 20.5 (17.4-23.9) | 19.3 (15.5-22.8) | <0.01 |  | 31.2 (24.8, 40.0) | 30.0 (23.7, 39.3) | 0.08 |
| Comorbidities, n (%) |  |  |  |  |  |  |  |
| Diabetes mellitus | 16 (2.6) | 31 (9.2) | <0.01 |  | 35 (2.4) | 33 (5.2) | <0.01 |
| Hypertension | 6 (1.0) | 6 (1.8) | 0.28 |  | 12 (0.8) | 15 (2.4) | <0.01 |
| Kidney disease | 1 (0.2) | 1 (0.3) | 1.00 |  | 5 (0.3) | 11 (1.7) | <0.01 |
| Sepsis | 5 (0.8) | 9 (2.7) | 0.04 |  | 1 (0.1) | 0 (0) | 1.00 |
| Cardiac failure | 27 (4.4) | 15 (4.5) | 0.95 |  | 4 (0.3) | 2 (0.3) | 1.00 |
| Respiratory failure | 5 (0.8) | 2 (0.6) | 1.00 |  | 6 (0.4) | 1 (0.2) | 0.68 |
| No. of nephrotoxicity drugs, n (%) |  |  |  |  |  |  |  |
| ≥3 | 173 (28.0) | 80 (23.8) | 0.16 |  | 300 (20.4) | 142 (22.2) | 0.35 |
| Renal function after contrast exposure, M (IQR) |  |  |  |  |  |  |  |
| eGFR (ml/min/1.73㎡) | 107.7 (90.8, 128.5) | 116.9 (96.5, 135.6) | <0.01 |  | 136.1 (116.0, 158.8) | 139.7 (116.6, 162.1) | 0.08 |
| SCr examination time after contrast exposure (days) | 3.7 (1.9, 5.6) | 2.8 (1.1, 4.8) | <0.01 |  | 3.3 (1.6, 5.3) | 2.7 (0.9, 4.7) | <0.01 |
| SCr (μmol/L) | 21.5 (17.9, 25.6) | 19.8 (17.2, 24.2) | <0.01 |  | 32.0 (25.8, 40.2) | 30.6 (24.5, 39.5) | <0.01 |
| AKI (KDIGO), n (%) | 72 (11.7) | 32 (9.5) | 0.31 |  | 83 (5.7) | 31 (4.9) | 0.53 |
| AKI stage (KDIGO), n (%) |  |  |  |  |  |  |  |
| Stage 1 | 40 (6.8) | 18 (5.4) | 0.49 |  | 40 (2.7) | 39 (3.0) | 0.02 |
| Stage 2 | 18 (2.9) | 11 (3.3) |  |  | 11 (0.8) | 9 (1.4) |  |
| Stage 3 | 12 (1.9) | 3 (0.9) |  |  | 32 (2.1) | 3 (0.5) |  |

Abbreviations: No., Number; ICU, Intensive Care Unit; eGFR, estimated Glomerular Filtration Rate; SCr, Serum Creatine; AKI, Acute Kidney Injury; KDIGO, Kidney Disease: Improving Global Outcomes.

**Supplemental Table 6 Patients characteristics in subgroup by eGFR**

| Characteristic | Baseline eGFR≥60 ml/min/1.73m^2^ | | |  | Baseline eGFR<60 ml/min/1.73m2 | | |
| --- | --- | --- | --- | --- | --- | --- | --- |
|  | Exposure group | Control group | P |  | Exposure group | Control group | P |
| No. of patients | 2068 | 960 |  |  | 18 | 15 |  |
| Age (year), M (IQR) | 4.9 (1.4, 9.2) | 3.9 (1.2, 8.2) | <0.01 |  | 3.5 (0.4, 7.2) | 2.2 (0.3, 9.6) | 0.84 |
| Male, n (%) | 1167 (56.4) | 568 (59.2) | 0.15 |  | 14 (77.8) | 11 (73.3) | 1.00 |
| ICU admission, n (%) | 223 (10.8) | 82 (8.5) | 0.06 |  | 4 (22.2) | 2 (13.3) | 0.66 |
| Baseline renal function, M (IQR) |  |  |  |  |  |  |  |
| Baseline eGFR (ml/min/1.73m^2^) | 131.8 (110.2, 157.3) | 136.3 (112.8, 162.9) | <0.01 |  | 50.4 (45.9, 52.8) | 50.3 (39.9, 55.2) | 0.65 |
| Baseline SCr (μmol/L) | 27.0 (21.3, 35.9) | 25.0 (19.2, 34.1) | <0.01 |  | 74.7 (48.6, 96.1) | 75.3 (40.5, 98.9) | 0.91 |
| Comorbidities, n (%) |  |  |  |  |  |  |  |
| Diabetes mellitus | 51 (2.5) | 61 (6.4) | <0.01 |  | 0 (0.0) | 3 (20.0) | 0.08 |
| Hypertension | 17 (0.8) | 21 (2.2) | <0.01 |  | 1 (5.6) | 0 (0.0) | / |
| Kidney disease | 6 (0.3) | 12 (1.3) | <0.01 |  | 0 (0.0) | 0 (0.0) | / |
| Sepsis | 6 (0.3) | 9 (0.9) | 0.03 |  | 0 (0.0) | 0 (0.0) | / |
| Cardiac failure | 31 (1.5) | 16 (1.8) | 0.73 |  | 0 (0.0) | 1 (6.7) | 0.45 |
| Respiratory failure | 11 (0.5) | 3 (0.3) | 0.41 |  | 0 (0.0) | 0 (0.0) | / |
| No. of nephrotoxicity drugs, n (%) |  |  |  |  |  |  |  |
| ≥3 | 466 (22.5) | 217 (22.6) | 0.97 |  | 7 (38.9) | 5 (33.3) | 1.00 |
| Renal function after contrast exposure, M (IQR) |  |  |  |  |  |  |  |
| eGFR (ml/min/1.73㎡) | 128.4 (107.7, 151.8) | 132.0 (110.6, 156.3) | <0.01 |  | 57.8 (40.1, 96.7) | 60.8 (44.5, 73.3) | 0.95 |
| SCr examination time after contrast exposure (days) | 3.4 (1.7, 5.3) | 2.8 (0.9, 4.8) | <0.01 |  | 3.8 (0.8, 4.9) | 2.0 (1.5, 2.9) | 0.40 |
| SCr (μmol/L) | 28.6 (22.2, 37.2) | 26.2 (20.0, 34.4) | <0.01 |  | 61.3 (34.0, 91.6) | 59.8 (32.1, 89.2) | 0.97 |
| AKI (KDIGO), n (%) | 153 (7.4) | 63 (6.6) | 0.41 |  | 2 (11.1) | 0 (0.0) | / |
| AKI stage (KDIGO), n (%) | 81 (3.9) | 37 (3.9) |  |  |  |  |  |
| Stage 1 | 29 (1.4) | 20 (2.1) | 0.01 |  | 1 (5.6) | 0 (0.0) | / |
| Stage 2 | 43 (2.1) | 6 (0.6) |  |  | 0 (0.0) | 0 (0.0) |  |
| Stage 3 | 2068 | 960 |  |  | 1 (5.6) | 0 (0.0) |  |

Abbreviations: No., Number; ICU, Intensive Care Unit; eGFR, estimated Glomerular Filtration Rate; SCr, Serum Creatine; AKI, Acute Kidney Injury; KDIGO, Kidney Disease: Improving Global Outcomes.

**Supplemental Table 7 Results of multivariate logistic regression**

| Variable | Full population  (n=3061) | |  | Patients underwent CT  (n=1818) | |  | Patients underwent MRI  (n=1243) | |
| --- | --- | --- | --- | --- | --- | --- | --- | --- |
|  | OR (95%CI) | P |  | OR (95%CI) | P |  | OR (95%CI) | P |
| Contrast enhanced | 1.51  (1.09, 2.10) | 0.01 |  | 1.44  (0.96, 2.17) | 0.08 |  | 1.39  (0.78, 2.48) | 0.26 |
| Age (year) | 0.87  (0.84, 0.91) | <0.01 |  | 0.83  (0.79, 0.88) | <0.01 |  | 0.92  (0.87, 0.98) | <0.01 |
| Female | 1.01  (0.75, 1.35) | 0.96 |  | 1.00  (0.68, 1.45) | 0.98 |  | 1.10  (0.68, 1.77) | 0.69 |
| ICU admission | 1.47  (0.97, 2.22) | 0.07 |  | 1.65  (0.98, 2.76) | 0.06 |  | 1.02  (0.47, 2.20) | 0.97 |
| Baseline eGFR (ml/min/1.73m^2^) | 1.02  (1.01, 1.02) | <0.01 |  | 1.02  (1.01, 1.02) | <0.01 |  | 1.02  (1.01, 1.02) | <0.01 |
| No. of comorbidities |  |  |  |  |  |  |  |  |
| ≥1 | 2.97  (1.89, 4.65) | <0.01 |  | 4.02  (2.38, 6.82) | <0.01 |  | 1.19  (0.44, 3.21) | 0.73 |
| No. of nephrotoxicity drugs |  |  |  |  |  |  |  |  |
| ≥3 | 1.23  (0.88, 1.72) | 0.22 |  | 1.40  (0.90, 2.17) | 0.13 |  | 1.05  (0.61, 1.80) | 0.86 |

Abbreviations: No., Number; ICU, Intensive Care Unit; eGFR, estimated Glomerular Filtration Rate; SCr, Serum Creatine; AKI, Acute Kidney Injury.

**Supplemental Table 7 Continues**

| Variable | Age<2 years old  (n=954) | |  | Age≥2 years old  (n=2107) | |  | Baseline eGFR≥60 ml/min/1.73m^2^  (n=3028) | |  | Propensity-matched  (n=1844) | |
| --- | --- | --- | --- | --- | --- | --- | --- | --- | --- | --- | --- |
|  | OR (95%CI) | P |  | OR (95%CI) | P |  | OR (95%CI) | P |  | OR (95%CI) | P |
| Contrast enhanced | 1.89  (1.16, 3.08) | 0.01 |  | 1.29  (0.82, 2.02) | 0.27 |  | 1.49  (1.07, 2.07) | 0.02 |  | 1.55  (1.06, 2.27) | 0.02 |
| Age (year) | 0.64  (0.41, 0.99) | <0.05 |  | 0.94  (0.89, 0.99) | 0.03 |  | 0.87  (0.84, 0.91) | <0.01 |  | 0.83  (0.78, 0.88) | <0.01 |
| Female | 0.74  (0.47, 1.16) | 0.19 |  | 1.3  (0.87, 1.95) | 0.20 |  | 0.98  (0.73, 1.31) | 0.88 |  | 0.80  (0.54, 1.19) | 0.27 |
| ICU admission | 1.27  (0.72, 2.22) | 0.41 |  | 1.53  (0.81, 2.9) | 0.19 |  | 1.50  (0.99, 2.27) | 0.06 |  | 1.14  (0.66, 1.97) | 0.64 |
| Baseline eGFR (ml/min/1.73m^2^) | 1.02  (1.01, 1.03) | <0.01 |  | 1.02  (1.01, 1.02) | <0.01 |  | 1.02  (1.01, 1.02) | <0.01 |  | 1.02  (1.01, 1.02) | <0.01 |
| No. of comorbidities |  |  |  |  |  |  |  |  |  |  |  |
| ≥1 | 3.67  (2.11, 6.36) | <0.01 |  | 1.30  (0.5, 3.39) | 0.60 |  | 3.03  (1.93, 4.76) | <0.01 |  | 4.06  (2.27, 7.24) | <0.01 |
| No. of nephrotoxicity drugs |  |  |  |  |  |  |  |  |  |  |  |
| ≥3 | 1.07  (0.64, 1.77) | 0.81 |  | 1.39  (0.88, 2.19) | 0.16 |  | 1.21  (0.86, 1.70) | 0.27 |  | 1.33  (0.86, 2.06) | 0.20 |

Abbreviations: No., Number; ICU, Intensive Care Unit; eGFR, estimated Glomerular Filtration Rate; SCr, Serum Creatine; AKI, Acute Kidney Injury.

**Supplemental Table 8** **The distribution of children with SCr and diagnosed by AKI in 1-7 days after contrast agent exposure.**

| Time after contrast exposure | Exposure group (n=2086) | |  | Control group (n=975) | |
| --- | --- | --- | --- | --- | --- |
|  | N^a^ (%) | AKI (KDIGO), n (%) |  | N^a^ (%) | AKI (KDIGO), n (%) |
| Day 1 | 543 (15.7) | 34 (6.3) |  | 357 (22.8) | 12 (3.4) |
| Day 2 | 524 (15.2) | 32 (6.1) |  | 227 (14.5) | 17 (7.5) |
| Day 3 | 492 (14.3) | 21 (4.3) |  | 216 (13.8) | 20 (9.3) |
| Day 4 | 504 (14.6) | 31 (6.2) |  | 204 (13.0) | 15 (7.4) |
| Day 5 | 441 (12.8) | 26 (4.0) |  | 209 (13.4) | 14 (6.7) |
| Day 6 | 488 (14.1) | 33 (5.0) |  | 167 (10.7) | 12 (7.2) |
| Day 7 | 459 (13.3) | 43 (6.7) |  | 184 (11.8) | 15 (8.2) |
| Total^b^ | 3451 (100) | 220 (6.4) |  | 1564 (100) | 105 (6.7) |

Abbreviations: eGFR, estimated Glomerular Filtration Rate; SCr, Serum Creatine; AKI, Acute Kidney Injury; KDIGO, Kidney Disease: Improving Global Outcomes.

1. The “N” refers to the number of children with SCr within that day.
2. The “Total” refers to the sum of the number of children with SCr per day.


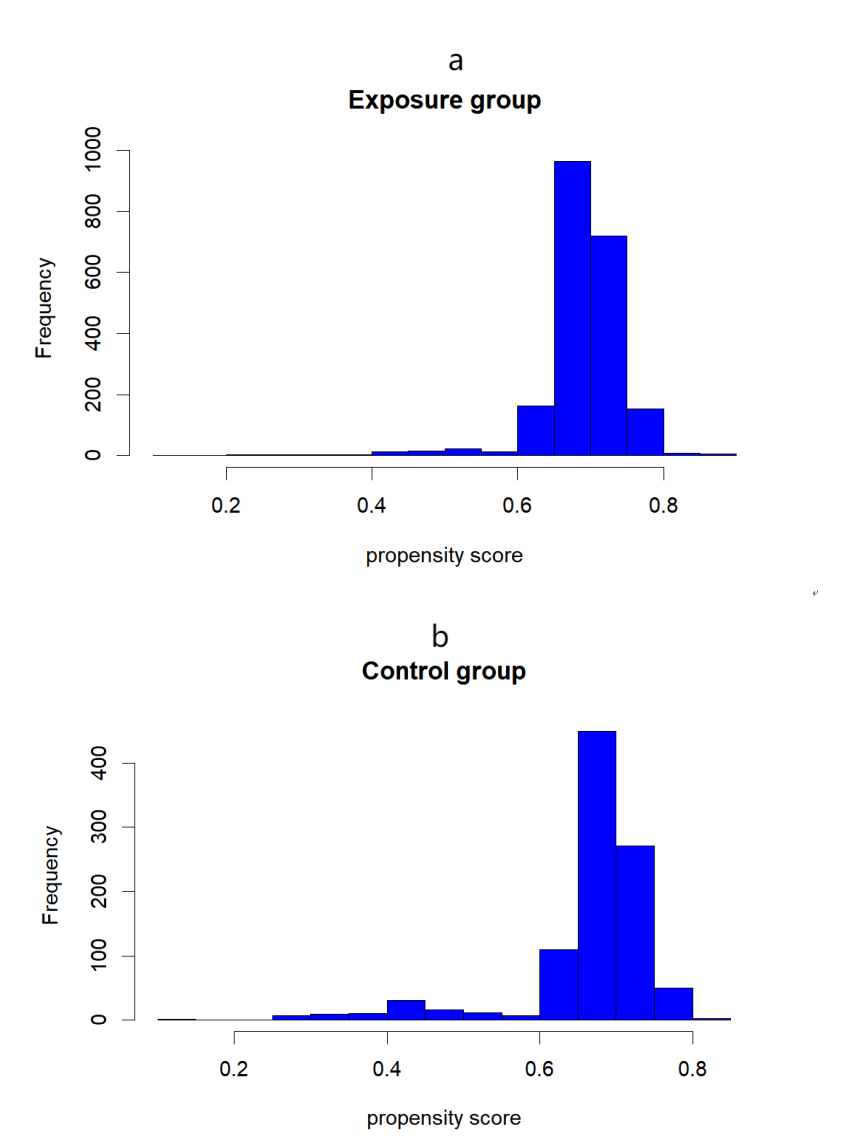


**Supplemental Figure 1** **Distribution of propensity score in exposure group (a) and control group (b).**


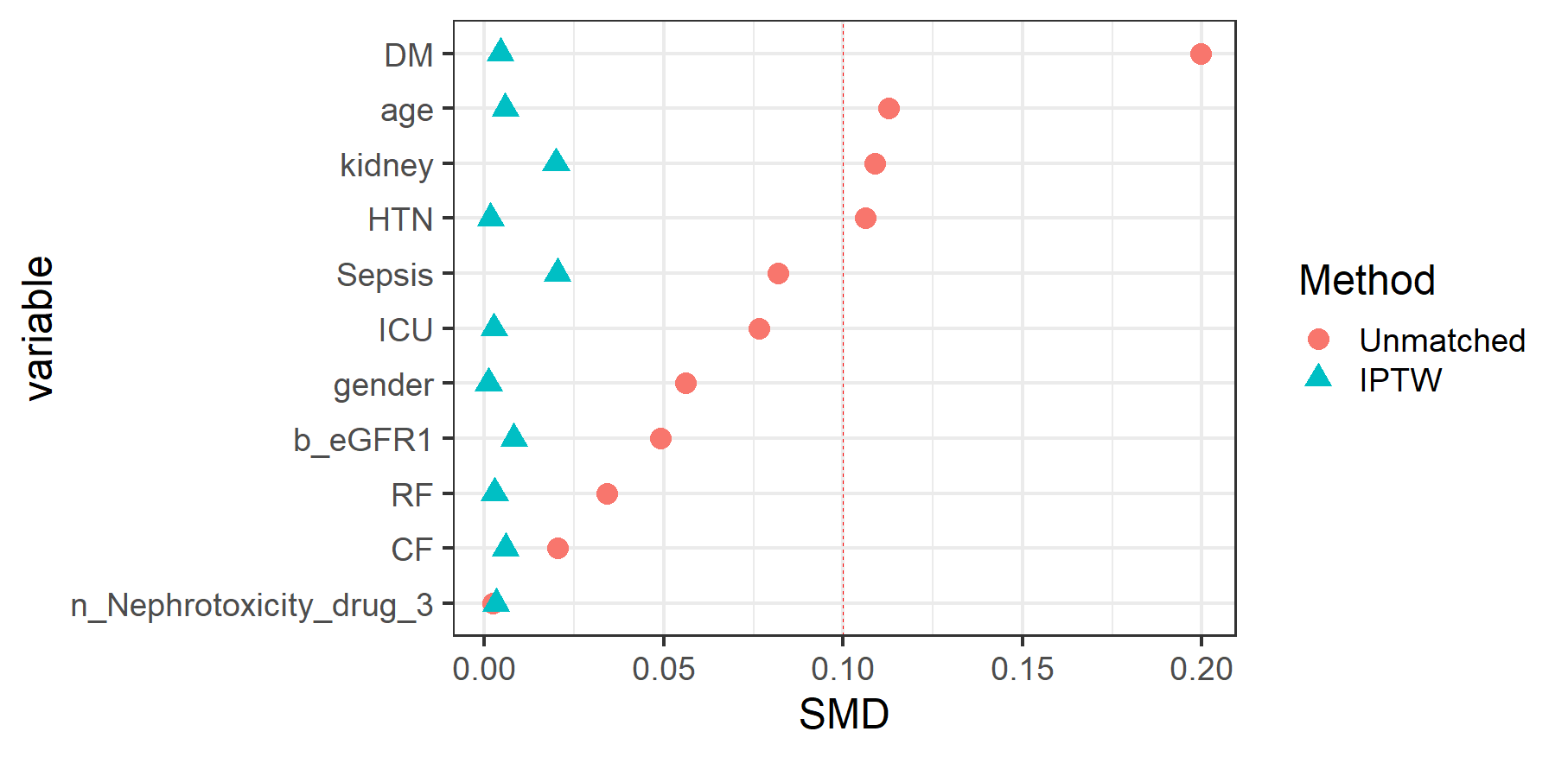


**Supplemental Figure 2 Standardized mean differences (SMDs) of different variables between the exposure group and the control group in unmatched population and inverse probability of treatment weighting (IPTW) cohort.**


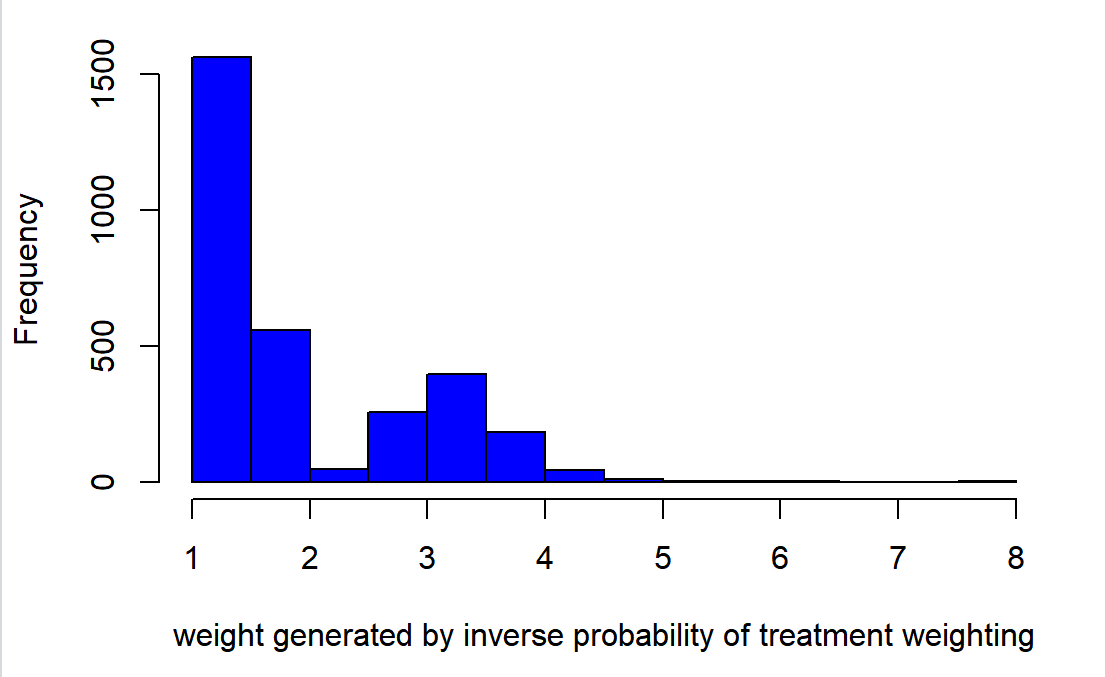


**Supplemental Figure 3 Distribution of weights generated by inverse probability of treatment weighting.**


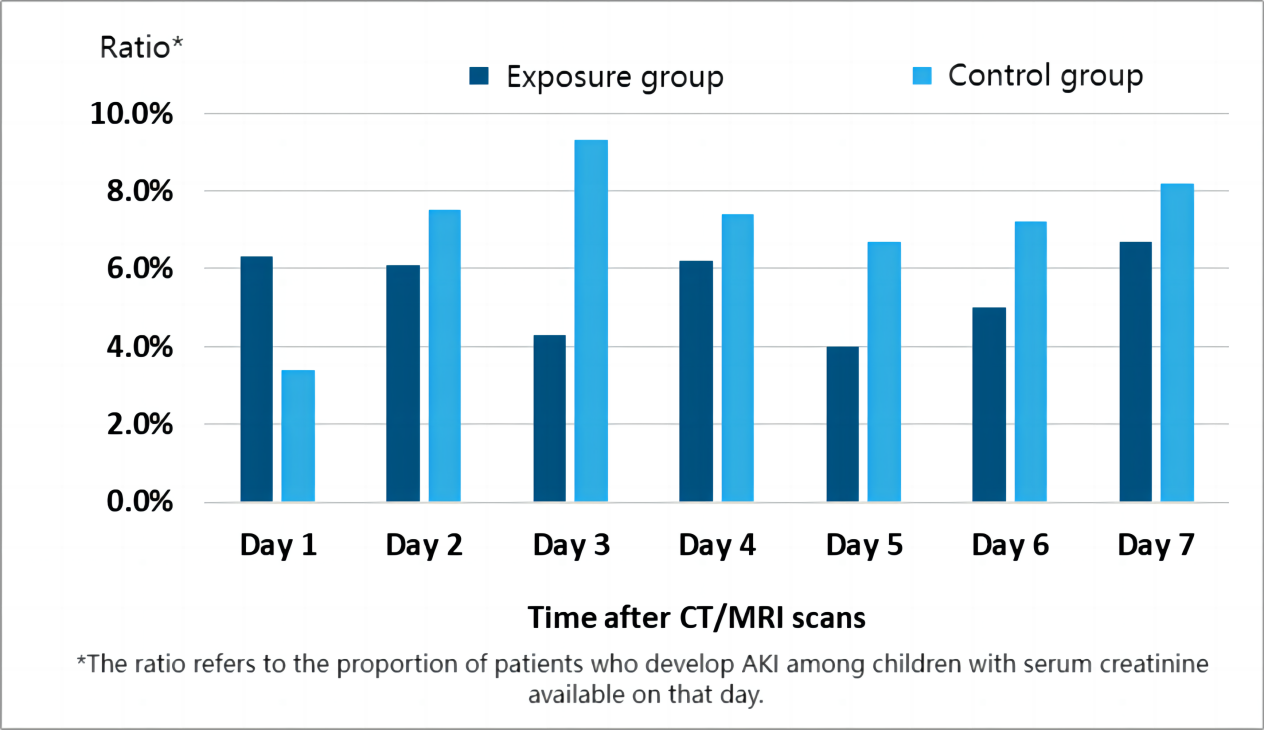


**Supplemental Figure 4 The distribution of children diagnosed with AKI in 1-7 days after contrast agent exposure.**
